# Dual Chaperone Role of the C-Terminal Propeptide in Folding and Oligomerization of the Pore-Forming Toxin Aerolysin

**DOI:** 10.1371/journal.ppat.1002135

**Published:** 2011-07-14

**Authors:** Ioan Iacovache, Matteo T. Degiacomi, Lucile Pernot, Sylvia Ho, Marc Schiltz, Matteo Dal Peraro, F. Gisou van der Goot

**Affiliations:** 1 Global Health Institute, Ecole Polytechnique Fédérale de Lausanne, Lausanne, Switzerland; 2 Institute of Bioengineering, Ecole Polytechnique Fédérale de Lausanne, Lausanne, Switzerland; 3 Laboratoire de Cristallographie, Ecole Polytechnique Fédérale de Lausanne, Lausanne, Switzerland; Yale University School of Medicine, United States of America

## Abstract

Throughout evolution, one of the most ancient forms of aggression between cells or organisms has been the production of proteins or peptides affecting the permeability of the target cell membrane. This class of virulence factors includes the largest family of bacterial toxins, the pore-forming toxins (PFTs). PFTs are bistable structures that can exist in a soluble and a transmembrane state. It is unclear what drives biosynthetic folding towards the soluble state, a requirement that is essential to protect the PFT-producing cell. Here we have investigated the folding of aerolysin, produced by the human pathogen *Aeromonas hydrophila*, and more specifically the role of the C-terminal propeptide (CTP). By combining the predictive power of computational techniques with experimental validation using both structural and functional approaches, we show that the CTP prevents aggregation during biosynthetic folding. We identified specific residues that mediate binding of the CTP to the toxin. We show that the CTP is crucial for the control of the aerolysin activity, since it protects individual subunits from aggregation within the bacterium and later controls assembly of the quaternary pore-forming complex at the surface of the target host cell. The CTP is the first example of a C-terminal chain-linked chaperone with dual function.

## Author Summary

Many pathogenic bacteria produce proteins, called pore-forming toxins, designed to perforate the plasma membrane of target cells thus perturbing host cell integrity and functionality. It is, however, important that these toxins do not form pores in the producing bacterium. To prevent this, bacteria initially produce them in a soluble state. After being secreted by the bacterium, the toxin subsequently acquires – often through a multimerization step– the ability to insert into the membrane. Here we were interested in the mechanisms ensuring that the toxin initially folds into the soluble state. Using as an example aerolysin from the human pathogen *Aeromonas hydrophila*, we show that the bacterium produces the toxin with a C-terminal extension of about 45 amino acids that promotes the folding of the protein into the soluble state. We find that by mutating or removing this extension, the protein folds poorly or not at all. Addition of the peptide *in trans* however lead to partial recovery of activity suggesting that this extension promotes folding, and being intramolecular thus results in a very high effective concentration. In addition to this chaperone role for correctly folding the monomeric form of the toxin, the C-terminal peptide is also crucial for controlling the folding of the quaternary structure of the mature pore complex at the surface of the target host cell.

## Introduction

Many organisms, as diverse as bacteria, parasites, sea anemones or plants, produce membrane damaging proteins to protect themselves or to modify the behavior of their host [1]. Amongst these pore-forming proteins (PFPs), we find bacterial pore-forming toxins (PFTs). These are produced as soluble proteins that diffuse and bind to target cells via specific receptors. Many subsequently assemble into ring-like structures [2], undergoing a conformational change with consequent exposure of hydrophobic surfaces. This drives spontaneous membrane insertion, leading to the formation of water filled pores.

**Figure 1:**
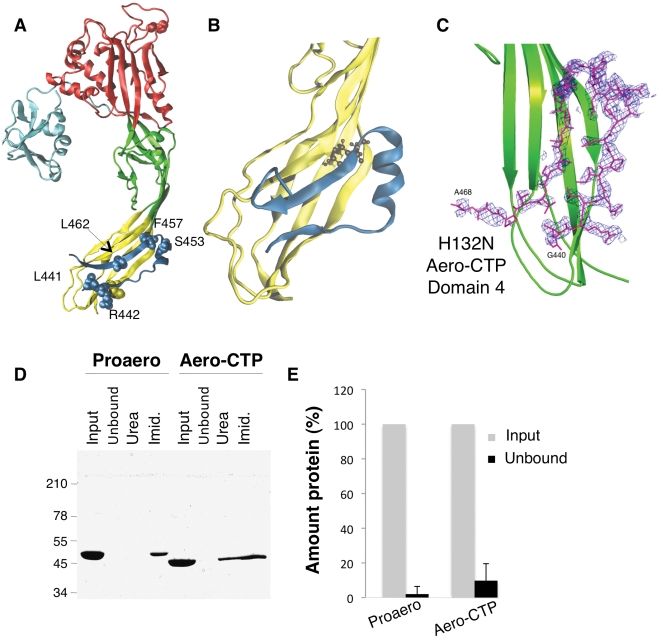
The CTP remains bound to aerolysin following trypsin cleavage. **A: **Structure of the proaerolysin monomer as seen in the dimeric soluble form of the toxin (1PRE). Structural domains are color coded with Domain 1 (light blue) and the large lobe of the protein additionally divided into three domains, Domain 2 (red), Domain 3 (green) and Domain 4 (yellow). The C-terminal peptide (CTP) is shown in dark blue and the different residues examined in this study are represented in space- filled model. **B:** Snapshot of Domain 4 with CTP after 100 ns of MD simulation. The main body of Domain 4 is shown in yellow and the CTP in blue. **C**: Close-up of the structure of the trypsinized H132N (pdb 3G4O) showing the *F*_obs_-*F*_calc_ electron density map of Domain 4 (in green) obtained prior to the reconstruction of the CTP. The map colored in blue is calculated at 2.3 Å resolution and contoured at a level of 2.0Å. The final model of the B subunit of the aerolysin mutant H132N is superimposed. **D:** Proaerolysin in 20 mM MES buffer pH5, 150 mM NaCl was adjusted to pH 8 by addition of 1 M Tris buffer pH 8.7 to avoid oligomerization and subsequently was processed with insoluble agarose trypsin beads. Unprocessed and processed toxins were incubated with His-bind resin preloaded with Nickel and incubated at 4°C for 30 minutes to allow binding of the protein and then extensively washed. The bead sample (input) was then split in two: half was treated with 4 M Urea (Urea) and the other half with 250 mM Imidazole (Imid.). After spinning down the beads, the supernatants were analyzed by SDS-PAGE followed by Coomassie blue staining. **E**: Coomassie blue stained gels, as in C, were quantified for 3 independent experiments using ImageJ. Error bars represent standard deviations.

This peculiarity of PFTs, and PFPs in general, raises two interesting questions. The first is: since PFPs can adopt two quite different conformations, how is the folding reaction during biogenesis directed towards obtaining the soluble fold? The second question is: what mechanisms prevent pore-formation from occurring in the producing cell? To address these related questions, we have chosen the PFT aerolysin (for review see [2]). Aerolysin is produced by the human pathogen *Aeromonas hydrophila* as an inactive precursor called proaerolysin. Conversion of proaerolysin to aerolysin involves proteolytic cleavage of a flexible 43-residue loop near the C-terminus (Figure 1A and Figure S1A). Maturation occurs after secretion from the bacterium and processing by gut enzymes or proteases present at the target cell surface [3], [4]. Since cleavage is essential for pore formation, it has been proposed that the role of the 43-residue C-terminal peptide (CTP) is to prevent premature oligomerization by steric hindrance, particularly within the producing bacterium.

Our aim was to address the precise role of the CTP by combining computational techniques, site-directed mutagenesis, structural analysis, and functional assays. Our study reveals that the CTP drives the protein into the soluble state during biogenesis, protecting proaerolysin from aggregation possibly by promoting folding, a quite unexpected observation considering the C-terminal location of the peptide. Interestingly, mutagenesis of specific residues in the CTP not only affected the efficiency of proaerolysin folding both *in vitro* and *in vivo*, but also reduced the capacity of the CTP to prevent premature assembly of the heptamer, highlighting the dual role of the CTP in 1) preventing aggregation of the newly synthesized protein possibly by assisting folding, and 2) controlling the quaternary assembly of the active complex.

## Results

### Covalent bonding is not required for binding of the CTP to aerolysin

The crystal structure of proaerolysin has been solved at 2.8 Å resolution [5]. The protein is L-shaped, composed of a continuous globular N-terminal domain (Figure 1A), Domain 1, and an elongated region consisting of three discontinuous structural domains (Figure 1A and Figure S1A). Domains 1 and 2 are involved in binding to cell surface receptors [6]. Domain 3 is involved in oligomerization and contains a loop that traverses the membrane upon pore formation [7]. Domain 4 has no known function but contains the CTP, which is folded as 2 anti-parallel β-strands connected by a short α-helix (Figure 1A).

To characterize the molecular interactions between the CTP and Domain 4, we performed classical molecular dynamics (MD) simulations. MD aims at quantitatively describing structural and thermodynamic properties of biomolecules within physiological-like conditions [8]. The existing potential energy models used in MD have been shown to provide accurate representation of atomistic interactions, and they have been used to investigate the biophysical properties of a broad variety of molecular systems, including the PFTs α-hemolysin from *S. aureus* [9], [10]. To remain as close as possible to experimental/*in vivo* conditions, we performed in MD simulations at room temperature (27°C) and atmospheric pressure (1 atm), and the proteins were solvated by water molecules at physiological salt concentration. MD simulations reported here were all based on the X-ray structures of wild type (WT) proaerolysin (for details about the structures used, see Methods). The loop connecting the CTP to the rest of the molecule is however not visible in any reported crystal structure of proaerolysin, probably due to its flexibility. Thus proaerolysin was *de facto* modeled in a situation mimicking a cleaved proaerolysin state (here after termed aerolysin-CTP). During the 200 ns of MD, the CTP remained firmly bound to the protein (Figure 1B). Native hydrogen bonds and salt bridges were preserved along the entire trajectory, as were secondary structure elements, both in the CTP and in Domain 4 (Figure 1B). The mean conservation of secondary structure in the system, i.e. the percentage of residues in a β-sheet conformation along the MD simulation with respect to the initial crystal structure, was 86±5% over the last 100 ns.

Since the MD simulations were performed with the absence of a covalent bond between the CTP and Domain 4, these observations pointed to a strong binding affinity of CTP for Domain 4. By computing electrostatic, van der Waals and solvation contributions to the binding of CTP to Domain 4, we estimated their binding energy to be 115 kcal/mol. Our MD-based observations thus suggest that the CTP remains bound to aerolysin upon proteolytic activation of the protoxin. This was confirmed using two independent experimental approaches. First, we determined the structure of the proteolytically processed form of an aerolysin mutant that is unable to form heptamers, namely H132N [11]. The crystal structure of the trypsin*-*processed H132N mutant was solved by molecular replacement to 2.3 Å resolution (PDB entry 3G4O) ([12], Protocol S1). Not only was the observed structure very similar to that of wild-type (WT) proaerolysin (root mean square deviation RMSD of 0.74 Å for subunits A and 0.92 Å for subunits B in the dimer), it also contained the CTP, in an essentially identical conformation (Figure 1C).

As a second approach to investigate whether the CTP remains bound to the mature toxin following proteolysis, we took advantage of our *E. coli* expressed WT proaerolysin, which harbors a six-histidine tag at the C-terminus, i.e. at the end of the CTP. When proaerolysin was incubated with Nickel beads, it remained attached to the beads as expected and could be eluted with imidazole but not with urea (Figure 1DE). When trypsin-processed toxin was incubated with Nickel beads, it also remained bound to the beads showing that the CTP had not been released upon proteolysis. Consistently with the non-covalent interaction between the mature toxin and the CTP, aerolysin could be released from the beads with urea (Figure 1D).

We had previously reported that processing of proaerolysin with trypsin leads to the release of the CTP from aerolysin [13]. This conclusion was based on the observation that fluorescence energy transfer was lost between a fluorescent probe, IEADANS, attached to an engineered cysteine on the CTP at position 445 and Trp-203 in Domain 4 [13]. Our current findings suggest that the previously observed release of the CTP was artefactually induced by the mutation and/or labeling of the cysteine at position 445. Indeed, in wild-type proaerolysin, Ile-445 on the CTP is buried within a hydrophobic pocket in Domain 4 and labeling of Cys-445 with the bulky and polar IAEDANS fluorophore (mimicked in Figure S1B) must have triggered a severe perturbation at the CTP-Domain 4 interface, leading to premature release of the CTP upon trypsin cleavage.

### Identification of key residues for CTP–aerolysin binding

**Figure 2:**
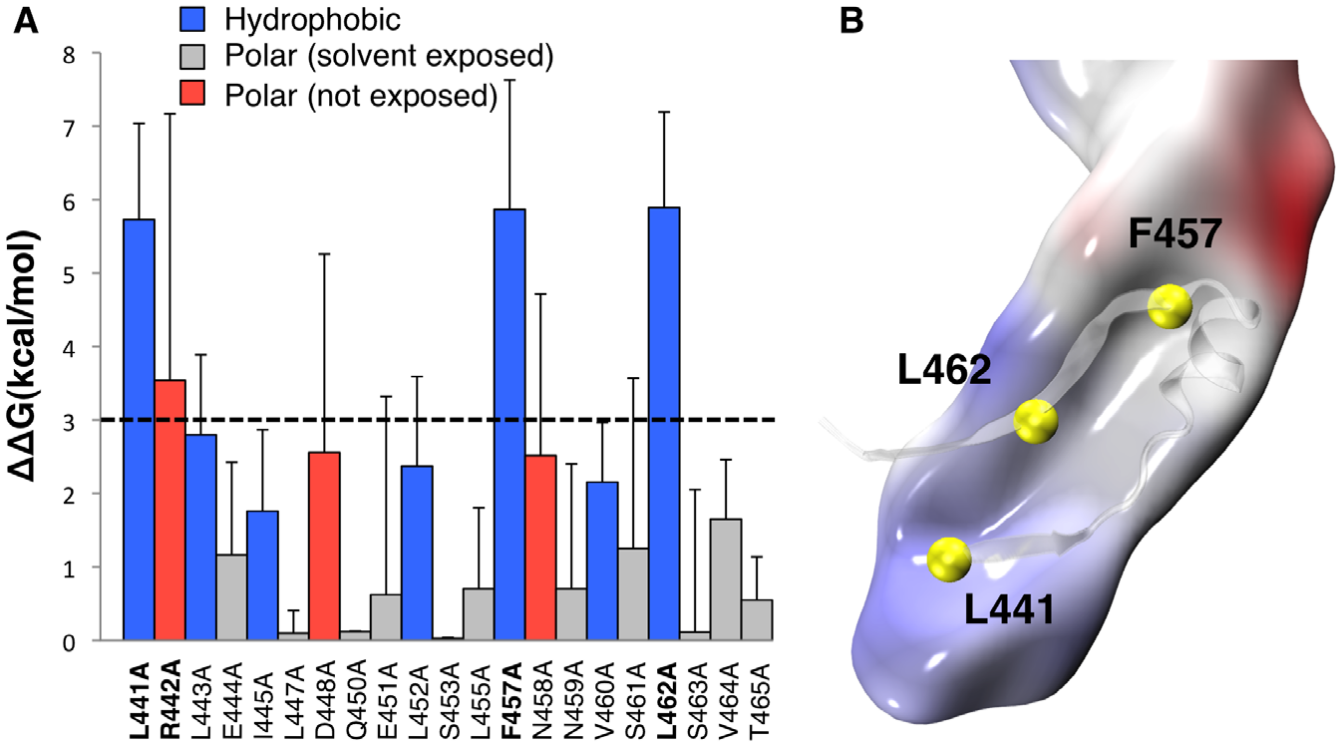
Molecular dynamics analysis of CTP binding. **A:** Residue-specific binding free energy obtained by MM-PBSA alanine scanning of CTP residues along a single 200 ns MD trajectory of the aerolysin-CTP system. Hydrophobic interactions are shown in blue, polar interactions in red and solvent exposed residues in grey. Higher standard deviations on solvent exposed residues Arg-442, Asp-448 and Asn-458 are due to the unstable nature of their binding and larger fluctuation during dynamics. **B**: Space-filled representation of a snapshot of Domain 4 showing the hydrophobic pocket in grey and the CTP residues involved in the hydrophobic interactions in yellow.

Both X-ray structures of WT proaerolysin and H132N aerolysin-CTP show the presence of a similar complex network of interactions between the CTP and Domain 4 composed of H-bonds (10 in subunit A, and 16 in subunit B), salt bridges (Asp-207 with Arg-442 and Lys-198 with Glu-451), and hydrophobic interactions. To identify key residues responsible for binding of the CTP to Domain 4, we performed *in silico* alanine scanning on most of the CTP. *In silico* mutation of a given CTP residue to alanine has the effect of removing most of the native non-bonded interactions (*i.e.*, electrostatic and van der Waals contributions) with the local environment. By comparing the binding free energy of the WT species and its alanine mutant, it is possible to estimate the individual contribution of a given CTP residues to the binding affinity with Domain 4. The greater the variation, the more the residue has a relevant role in the steady binding of the CTP to Domain 4. As expected, mutation of solvent exposed residues showed little variation in the binding free energy (Figure 2A). A low but significant variation was observed for certain polar residues, such as Asn-458, which forms a hydrogen bond with Asp-222, Asp-448, which forms a salt bridge with Lys-198, and especially Arg-442, which forms a salt bridge with Asp-207. The most dramatic variations in the binding free energy (∼6 kcal/mol) were observed for three hydrophobic residues: Leu-441, Phe-457 and Leu-462. All three residues point inside a hydrophobic pocket in Domain 4 underlying the CTP (Figure 2B). More specifically, Leu-441 interacts with Val-285, Ala-204, Pro-283 on Domain 4 and Leu-443 on the CTP, Leu-462 interacts with Val-217, Leu-219, and Ile-296 on Domain 4 and Ile-414 and Leu-443 on the CTP, and finally Phe-457 points straight into Domain 4, and is blocked by steric hindrance with Val-197 and Leu-297 on the Domain 4 and Ala-411 and Leu-452 on the CTP.

### Mutation of Phe-457 to Gly affects both the CTP and Domain 4

**Figure 3:**
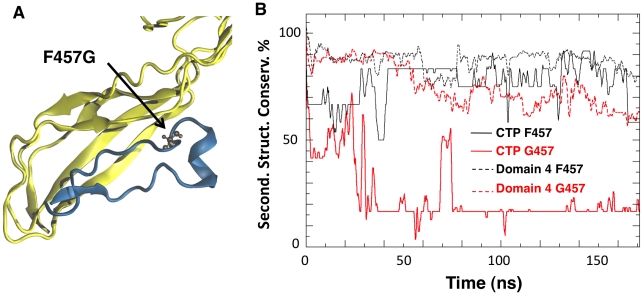
Mutation of Phe-457 affects the structure of the CTP and Domain 4. **A:** Representative snapshot of Domain 4 and CTP interactions in the F457G mutant system after 100 ns of MD simulations. Residue 457 is shown in ball-and-stick representation. **B**: The percentage of secondary structure conservation for the CTP (solid lines) and for Domain 4 (dashed lines) is reported. Conservation is higher for WT system (black) than for the F457G mutant (red).

Since Phe-457 on the CTP points straight into Domain 4, we investigated the effect of mutating this residue to glycine *in silico* using an MD setup similar to the one adopted for the WT species (Video S1). The mean conservation of the secondary structure of the CTP drastically dropped from 76±12% for the wild type to 17±4% for the F457G mutant (Figure 3B). The CTP structure remaining after the simulation was a portion of the α-helix (Figure 3A), which we determined to be the most stable structural element in an MD simulation of just the CTP in water (Figure S2BC). Interestingly, the F457G mutation also affected the underlying Domain 4 (Figure 3). Indeed, after 100 ns of MD, the mean conservation of the secondary structure of Domain 4 (including CTP) was 86±5% for the wild type and 67±5% for the F457G mutant. By computing electrostatic, van der Waals and solvation contributions to the binding of the mutated CTP (F457G) to domain 4, we estimated their binding free energy to be 75 kcal/mol. This represents a significant reduction with respect to the 115 kcal/mol previously estimated from the aerolysin-CTP MD simulation.

**Figure 4:**
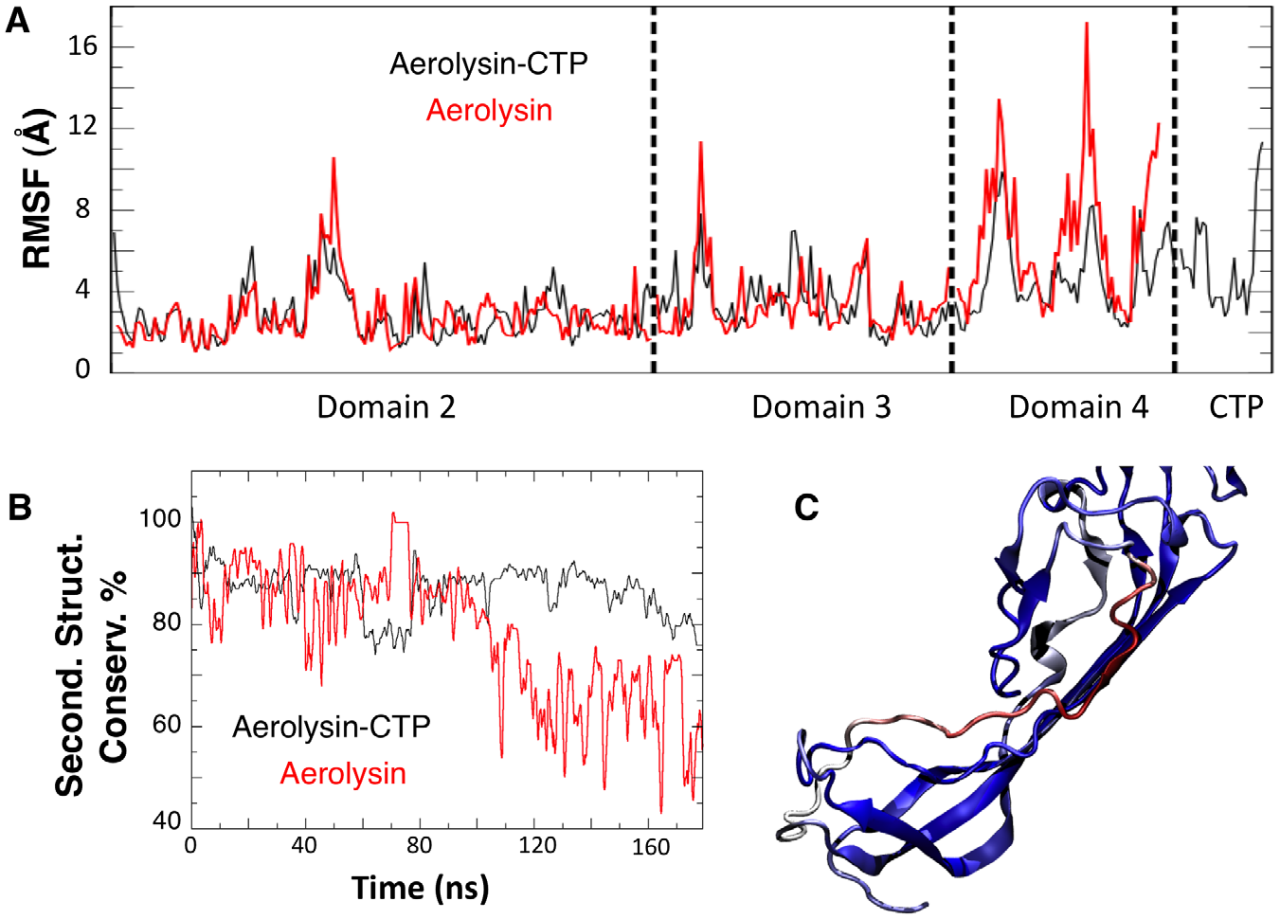
Removal of the CTP results in partial unfolding of Domain 4. **A:** Structural fluctuation per residue (calculated as RMSF) during the MD simulations of WT aerolysin with and without CTP. **B**: Comparison of the secondary structure conservation ratio for Domain 4 with (black) and without (red) the CTP. **C**: Representative snapshot of Domain 4 in WT aerolysin in the absence of the CTP after 100 ns of MD simulation. The coloring scale reports the degree of disorder as estimated using a set of disorder predictors (blue: mostly ordered; red: mostly disordered, see also Protocol S1).

The effects produced by a mutated CTP on Domain 4 prompted us to compare the structural features of aerolysin with and without its CTP. *In silico,* we removed the CTP from the proaerolysin crystal structure, and 200 ns MD simulation was performed (Video S2). Simulations performed in the presence and absence of the CTP were subsequently compared. The structural flexibility of each residue was quantified by calculating the root mean square fluctuation (RMSF) of the residue along the MD trajectory. Removing the CTP had no significant effect on the structure of Domain 2 and 3 (Figure 4A, Domain 1 was omitted from the simulation since it is known to act as an independent folding unit [14]). In contrast, removal of the CTP led to an average increase of 6.8±3.2 Å of the RMSF for a given residue in Domain 4 (Figure 4A), suggesting that the CTP stabilizes the structure of Domain 4. The CTP also had an influence on the secondary structure of Domain 4. This was assessed by tracking the percentage of secondary structure conservation along the two simulations, i.e. the percentage of residues adopting a ß-sheet conformation in the absence of CTP as compared to crystal structure of proaerolysin. In the absence of the CTP, the secondary structure conservation of Domain 4 was 67±10% (Figure 4B), compared to 86±5% in the presence of CTP, and the RMSD (root mean square fluctuation deviation) after 200 ns of MD was 8.8 Å, compared to 3.6 Å in the presence of CTP.

*In silico* removal of the CTP led to the unfolding of the β-strand encompassing residues Ser-272 to Ser-280 in Domain 4 (Figure 4C). Interestingly, a further sequence-based analysis using order prediction algorithms identified the 268-282 segment as the most disordered region of Domain 4 (for algorithms used see Protocol S1) (Figure S2A), raising the possibility that the ß-structure observed for this segment in the proaerolysin crystal structure is in fact imposed by the CTP. It is interesting to note that induced folding of intrinsically unstructured elements often involves hydrophobic, rather than polar, interactions [15] as observed here for the CTP-Domain 4 interface.

### Mutation or removal of the CTP prevents folding of aerolysin *in vivo* and *in vitro*

**Figure 5:**
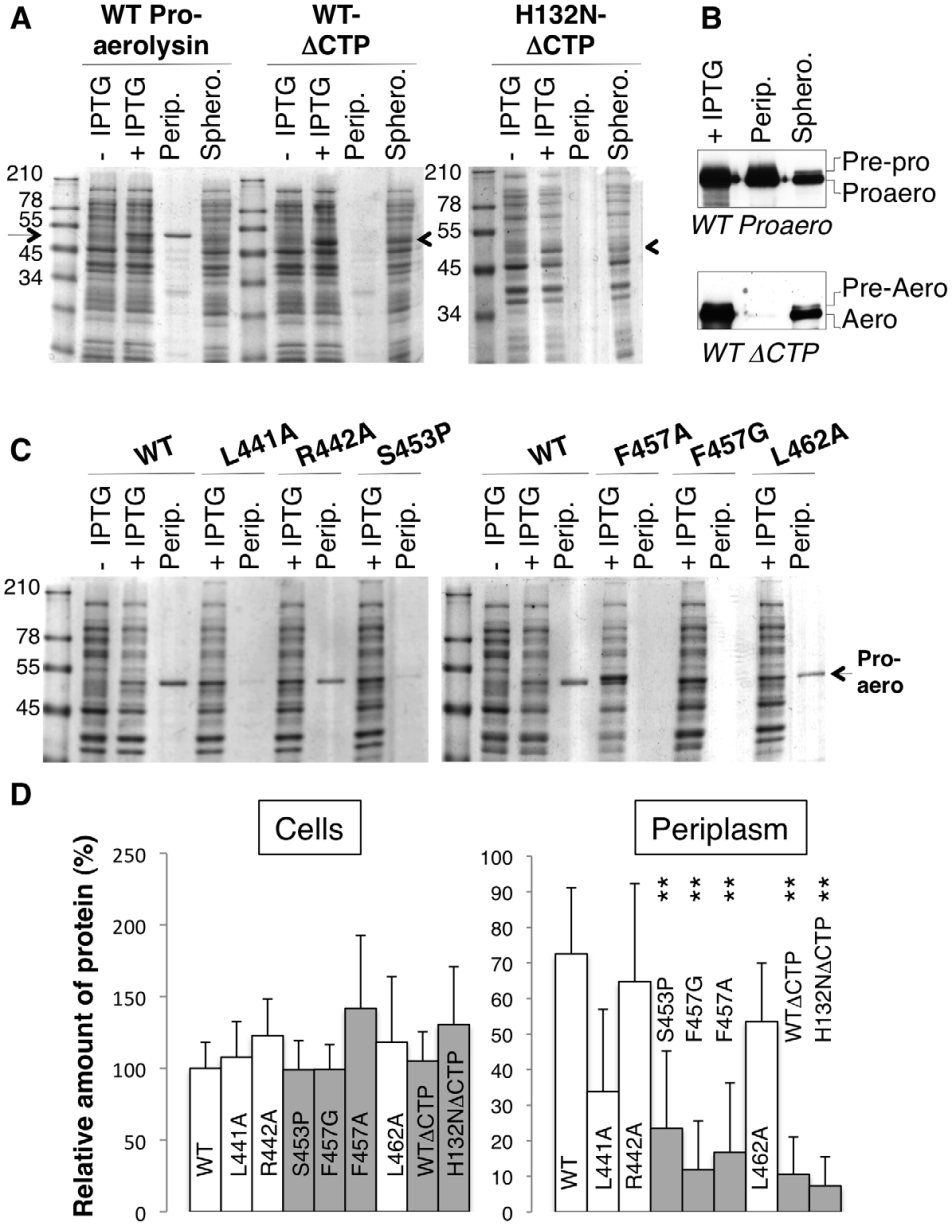
Removal or single point mutations of the CTP affect the folding of aerolysin *in vivo*. **A**: WT and H132N aerolysin, with and without the CTP, were expressed in the periplasm of *E. coli* upon induction with IPTG. Cell extracts were analyzed by SDS-PAGE and Coomassie blue staining before and after induction, as were the periplasmic (perip.) and the remaining spheroplast (sphero.) fractions. **B**: WT toxin, with and without the CTP, was expressed in the periplasm of *E. coli* upon induction with IPTG. Cell extracts, periplasmic and spheroplast fractions were analyzed by western blotting against aerolysin, revealing the signal peptidase dependent processing of signal peptide harboring precursors. **C**: WT and mutant proaerolysin were expressed in the periplasm of *E. coli*. Bacterial extracts and periplasmic fractions were analyzed by SDS-PAGE and Coomassie blue staining as in A. **D**: The amount of toxin present in cell extracts after IPTG induction as well as in the periplasmic fraction were quantified for 3 independent experiments using ImageJ (n = 3). Error bars represent standard deviations. Periplasmic toxin was normalized to the amount of toxin in the cell extracts. **: p<0.005.

The above MD analyses suggest that the structure of Domain 4 strongly depends on interactions with the CTP. This raised the interesting possibility that the CTP acts as a stabilizer or an intramolecular chaperone during biosynthetic folding of proaerolysin. A prediction from this hypothesis is that when synthesized and translocated across the inner E. *coli* membrane, aerolysin, i.e. lacking the CTP, should not be able to reach a soluble form in the bacterial periplasm. To test this, we generated constructs encoding only the N-terminal signal sequence and the mature protein without the CTP. Constructs were generated to express the WT version as well as H132N as to avoid potential oligomerization in the periplasm. As mentioned earlier, residue His-132 is required for heptamer formation [11]. When extracts from bacteria harboring WT or H132N aerolysin-ΔCTP expression constructs were analyzed by SDS-PAGE and Coomassie blue staining, a band migrating at the expected ≈50 kDa molecular weight was observed upon induction with IPTG, showing that bacteria were able to produce aerolysin-ΔCTP and that the protein was not degraded by periplasmic proteases (Figure 5 AD). When bacteria were processed to generate periplasmic and spheroplast fractions, proaerolysin, which migrates at ≈52 kDa, was recovered in the periplasmic fraction. In contrast WT or H132N variants of aerolysin-ΔCTP were only recovered in the spheroplast fraction (Figure 5 AD). Care was taken to induce toxin expression with a low IPTG concentration (0.25 mM) at a bacterial density of OD_600 nm_  = 0.6 for only 2 hrs at 18°C, to avoid jamming of the translocation machinery across the inner membrane and impair periplasmic folding. That periplasmic translocation of both proaerolysin (Figure 5B upper panel) and aerolysin-ΔCTP (Figure 5B lower panel) did occur under these conditions was confirmed by Western blot analysis. A weak band corresponding to pre-proaerolysin, i.e. protein for which signal sequence cleavage had not yet taken place, could be observed in the spheroplast fractions (Figure 5B). The bulk of both proaerolysin and aerolysin-ΔCTP however underwent processing by signal peptidase confirming that both forms of the toxin were properly translocated across the inner membrane (Figure 5B).

Our *in silico* alanine scanning analysis (Figure 2A) predicted that mutation of Leu-441, Phe-457 and Leu-462, and to a lesser extent Arg-442, to alanine should affect binding of the CTP to Domain 4. To test these MD-based predictions, we generated constructs to express these mutants in the *E. coli* periplasm. We also sought a mutation that would affect the secondary structure of the CTP but not the binding. We chose to change Ser-453 to proline since this residue localizes to the middle of the α-helix of the CTP (Figure 1A) and does not make contacts with Domain 4. In agreement, *in silico* mutation of Ser-453 to alanine did not lead to a significant variation in the binding free energy (Figure 2A). Due to the folding of its side chain back onto the protein backbone, proline imposes severe constraints to the backbone geometry leading to helix breaking.

**Figure 6:**
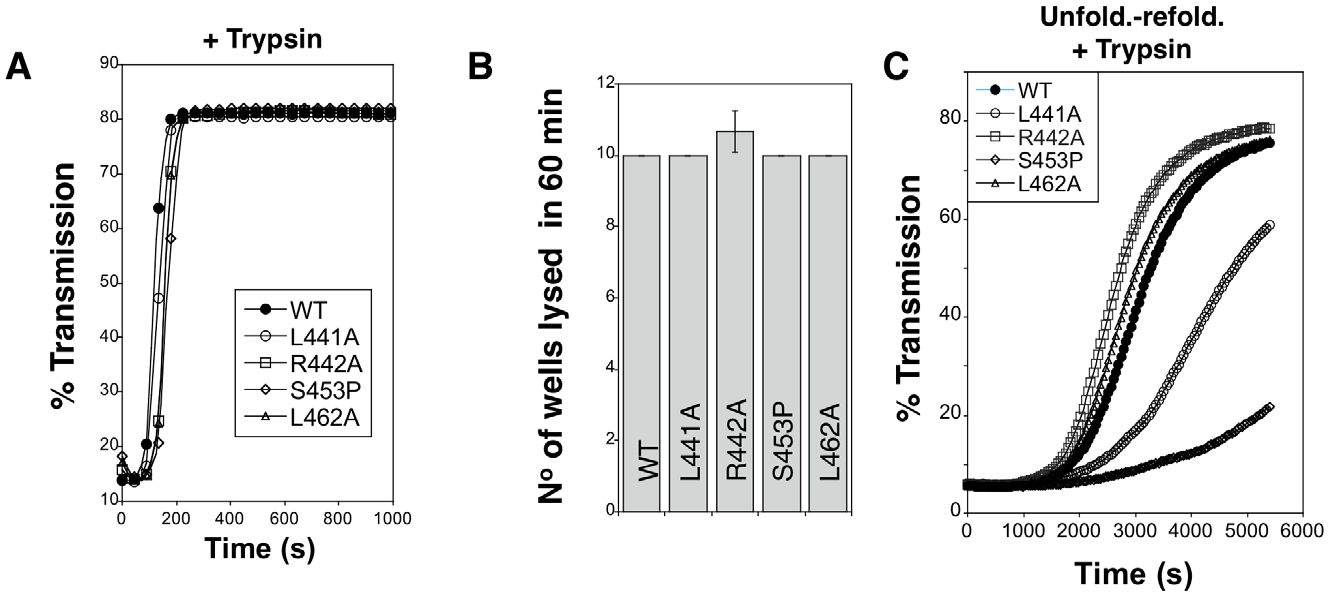
Hemolytic activity of WT and mutant aerolysins. **A.** WT and mutant proaerolysins were processed with soluble trypsin, added to erythrocytes at 20 µg/mL and the transmitted light at 600 nm of the sample was followed at room temperature as a function of time. Plots represent the percentage of transmitted light as recorded by the SpectraMax ME as a function of time. This is a representative experiment out of 4 independent experiments. **B:** WT and mutant proaerolysins (2 µg/mL) were processed with soluble trypsin, then subjected to a serial dilution (1/2) in a 96 well plate and incubated with erythrocytes. The number of wells lysed after 60 min at room temperature was determined. Error bars represent standard deviations (n = 3). **C**: WT and mutant proaerolysins (2 µg/mL) were unfolded in 4 M urea for 2 hrs, diluted 10 fold in a urea free medium. After 10 min, samples were treated with trypsin for 10 min. Erythrocytes were added and the transmitted light at 600 nm of the samples was followed at room temperature as a function of time. This is a representative experiment out of 5 independent experiments.

All proaerolysin mutants were detected in bacterial extracts showing that they were synthesized and not degraded to any significant extent (Figure 5B). Proaerolysins L441A, R442A and L462A were recovered in significant amounts in the periplasmic fraction (Figure 5D). Proaerolysin S453P was barely detectable in the periplasmic fraction (Figs. 5CD), but following purification low amounts of the protein could be obtained. Proaerolysins F457A/G were essentially undetectable in the periplasmic fraction (Figs. 5CD) and neither could be recovered following purification on Nickel columns. These observations show that mutating Ser-453 to proline or Phe-457 to glycine induced aggregation of proareolysin in the bacterial periplasm, either due to the exposure of a hydrophobic patch or improper folding of part of the protein. The small amounts of toxin that could be purified for all mutants was however properly folded as indicated by the WT-like hemolytic activity of the mutants following trypsin cleavage (Figure 6AB).

We next investigated the *in vitro* folding of the mutant proaerolysins. For this, proaerolysins, WT and mutants, were unfolded in urea. We have previously shown that proaerolysin unfolding in urea or GdnHCl occurs in two steps, corresponding to the unfolding of Domains 2 to 4, followed by the unfolding of the highly stable Domain 1 [14] (Figure S3AB). All four proaerolysin mutants showed very similar urea unfolding curves (Figure S3C). Following unfolding in 4 M urea, refolding of proaerolysins was triggered by dilution in a urea-free buffer. The efficiency of folding was indirectly monitored by measuring the hemolytic activity of the refolded proaerolysins after proteolysis with trypsin (trypsin was added 5 min after dilution in the urea free medium). Hemolysis was followed as a function of time. Under these conditions, refolded L441A and S453P systematically showed a delayed hemolytic activity (Figure 6C). These experiments reveal that *in vitro* folding into a soluble state of the L441A and S453P proaerolysin mutants was impaired and the extent correlated with the ability of the mutants to fold into a soluble state *in vivo* (Figure 5).

Altogether, these computational analyses and experimental observations indicate that the CTP is required for proper folding of aerolysin and that both the structure of the CTP and its binding affinity to Domain 4 are important for proaerolysin to reach a soluble active state.

### The aerolysin CTP promotes folding to a soluble state both in *cis* and in *trans*

Since aerolysin without the CTP could not be purified from bacteria, we studied the folding of aerolysin by cleavage of proaerolysin with trypsin followed by unfolding in urea. Unfolding transitions occurred at similar concentrations of chaotropic agents whether proaerolysin was processed by trypsin or not, suggesting proaerolysin and aerolysin-CTP have similar stabilities.

**Figure 7:**
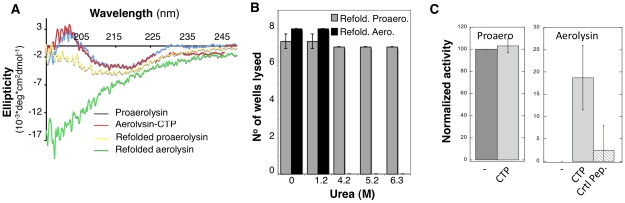
The CTP is required for refolding of aerolysin *in vitro*. **A:** Circular dichroïsm spectra were measured between 195 and 250 nm in 20 mM Hepes, 50 mM NaF pH 8 at 20°C. The spectra were acquired for proaerolysin before (blue) and after processing with trypsin (red, labeled aerolysin-CTP). Proteins were unfolded in 4 M urea, refolded by 10 fold dilution in a urea free buffer and the spectra of Refolded proaerolysin (yellow) and Refolded aerolysin (which has lost its peptide by unfolding) (green) were acquired after 1 hr at room temperature. **B**: Proaerolysin was subjected to proteolysis (red) or not (blue), unfolded in between 0 M and 6.3 M urea for 2 hrs and allowed to refold in urea free buffer before the hemolytic activity was assessed. Proaerolysin was processed with trypsin after refolding. The results are the mean of 3 independent experiments. Error bars represent the standard deviation. **C**: Proaerolysin (0.5 mg/mL) was or not processed with trypsin agarose beads. Upon bead removal, the protein was unfolded in 4 M urea at room temperature for 12 hours. Refolding was performed by dilution into a urea free buffer in the presence or absence of a 5-fold molar excess of CTP or control peptide. The hemolytic capacity was determined by serial dilution of the toxins and incubation with erythrocytes for 24 hrs.

Refolding was initiated by dilution into a urea free buffer and the tryptophan emission spectrum was measured at different times for more than 24 hrs. The maximum emission wavelength of proaerolysin rapidly shifted from 344 nm, corresponding to the unfolded protein, to 336 nm, corresponding to that of native proaerolysin. Under refolding conditions, the maximum emission wavelength of aerolysin –which had presumably lost its CTP during unfolding– however failed to reach that of native aerolysin-CTP, indicating that refolding did not occur or was partial. This was confirmed by circular dichroïsm (CD) in the far UV, which allows monitoring of secondary structure. As described previously, proaerolysin and aerolysin-CTP have very similar far UV CD spectra (Figure 7A) [16]. Upon unfolding and refolding, the spectrum of proaerolysin was to a large extent recovered (Figure 7A). In contrast, the spectrum of aerolysin under refolding conditions showed typical features of a random coil, with a strong negative ellipticity in the 200 nm spectral region possibly due to protein aggregation (Figure 7A). Thus, aerolysin was unable to reach a soluble state *in vitro* confirming the *in vivo* experiments (Figure 5 AD). Interestingly, under refolding conditions, aerolysin did not fold into a molten globule-like structure, which has native like secondary structure (Figure 7A). That aerolysin failed to reach a native conformation *in vitro* was confirmed by the lack of hemolytic activity after refolding from >4 M urea, in contrast to proaerolysin which refolded properly even from >6 M urea (Figure 7B).

All of the above-described observations point towards a role of the CTP in promoting the folding of proaerolysin into a soluble protein during biosynthesis. We finally investigated whether the CTP could also act when added in *trans* during *in vitro* refolding of aerolysin. We found that addition of 5-fold molar excess of synthetic CTP led to a significant and reproducible recovery in hemolytic activity of aerolysin, whereas addition of an irrelevant peptide did not (Figure 7C). The fact that rescue was only partial is not surprising since having a covalently bound CTP, as in proaerolysin, greatly increases the effective concentration of the peptide, a situation that cannot be mimicked by ectopic addition of excess CTP.

### CTP prevents aerolysin oligomerization

**Figure 8:**
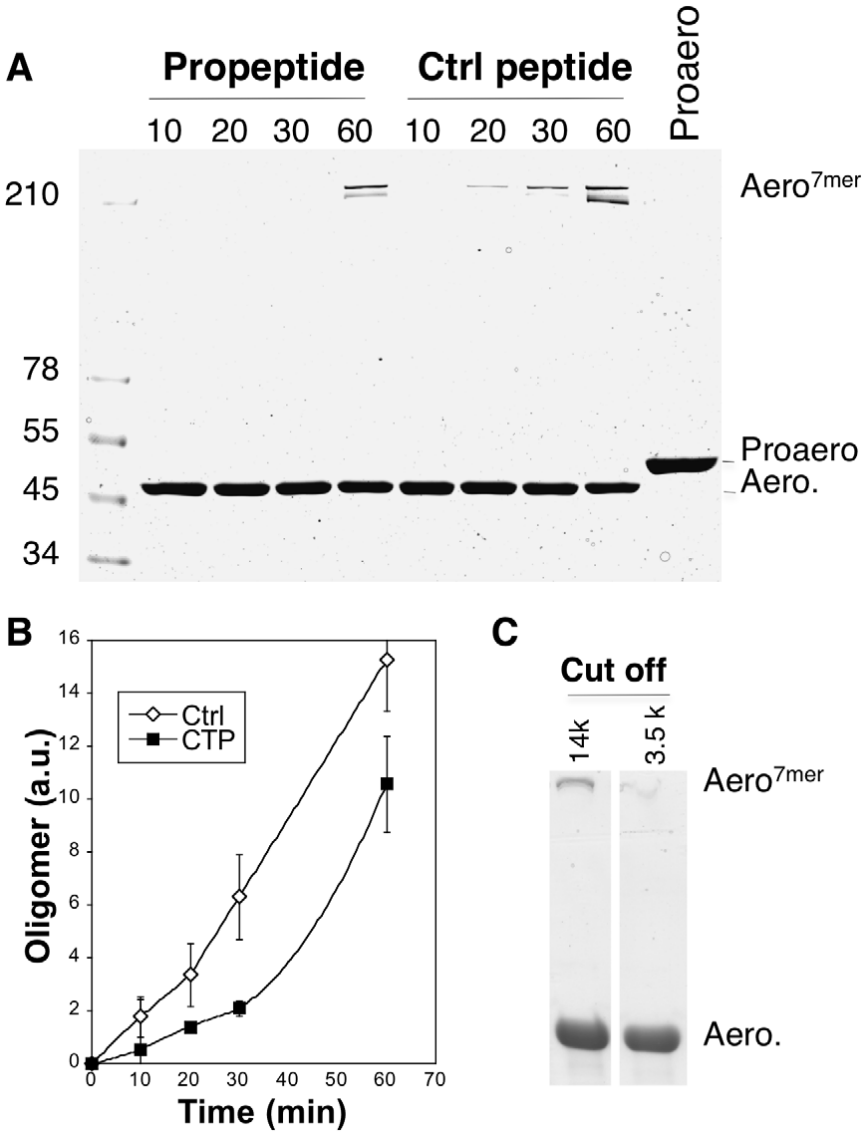
Aerolysin oligomerization is controlled by the CTP. **A:** Proaerolysin was activated with trypsin agarose beads at 4°C in a Tris pH 8 buffer to inhibit oligomerization and the sample was diluted with 50 mM Hepes pH 7 to a concentration of 10 µM in order to favor oligomerization in the presence or absence of a 5-fold molar excess (i.e. 50 µM) of synthetic CTP or control peptide. The sample was incubated at room temperature for different times and subsequently analyzed by SDS-PAGE followed by Coomassie blue staining. **B:** The amount of oligomer was quantified for 3 independent experiments using ImageJ (n = 3). Error bars represent standard deviations. **C:** Proaerolysin at a concentration of 1 mg/mL in a buffer at pH 8 (to inhibit oligomerization) was activated with trypsin agarose beads and subsequently dialyzed against 10 mM Hepes buffer pH 7.4, 10 mM NaCl to initiate the oligomerization process. Two different dialysis molecular weight cut offs where used: 14 kDa, which allows the passage of the CTPs and 3.5 kDa, which retains peptides the size of the CTP. After 2 hours of dialysis at RT, the samples were analyzed by SDS-PAGE followed by Coomassie blue staining.

It had previously been proposed for *Clostridium* α-toxin, a toxin with 27% sequence identity and 72% similarity to aerolysin, that the role of the CTP is to inhibit the oligomerization process [17]. We found that this role is also fulfilled by the aerolysin CTP. Proaerolysin was cleaved *in vitro* with trypsin and oligomerization was allowed to proceed in the absence or presence of a five-fold excess of either synthetic CTP or a control peptide. Heptamer formation was delayed by the presence of CTP (Figure 8AB). The role of the CTP in oligomerization was confirmed by the following observation. When proaerolysin was processed by trypsin in a pH 8 buffer to avoid oligomerization [11] and subsequently dialyzed against a neutral pH buffer to allow oligomerization, heptamer formation was only observed with a dialysis cut off that allowed the passage of the CTP (14 kDa) but not with a cut off that retained the CTP (3.5 kDa) (Figure 8C). Thus the binding of the CTP to Domain 4 inhibits oligomerization.

### Weaker CTP binding promotes oligomerization

**Figure 9:**
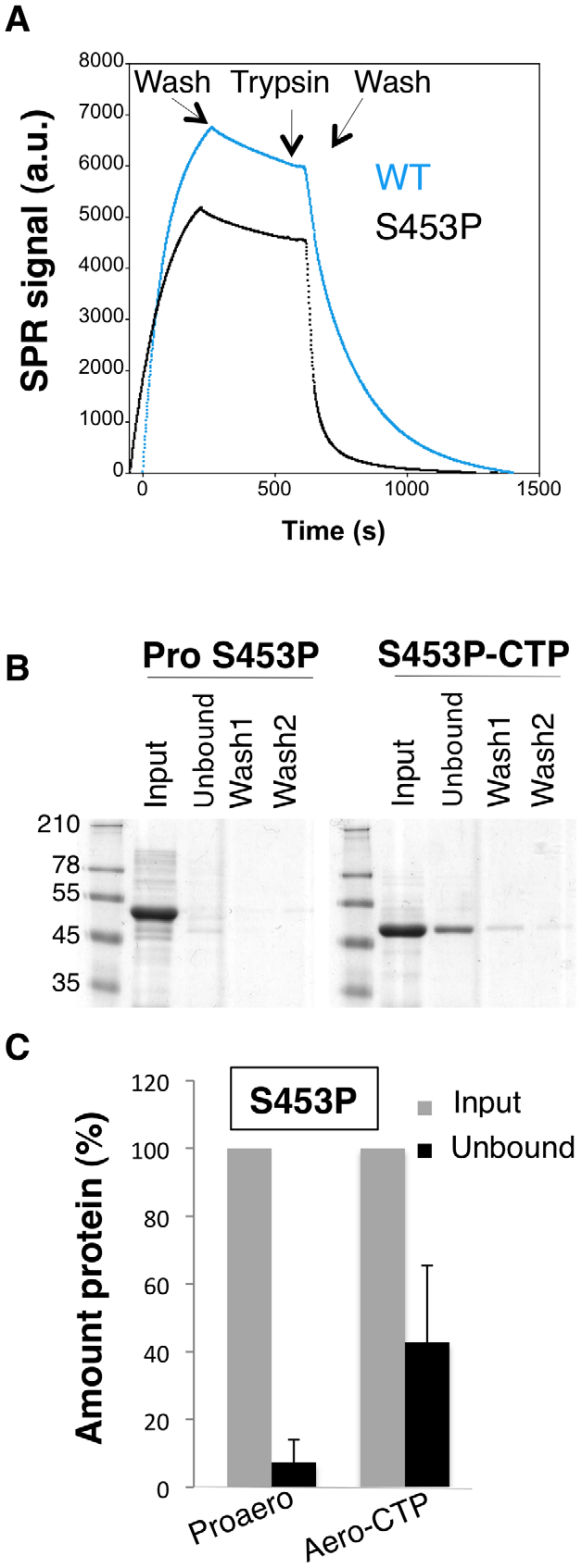
The S453P mutation leads to premature release of the CTP. **A**: Surface plasmon resonance analysis of mature toxin release following binding of His-tagged WT and S453P proaerolysin to Nickel charged sensor chip and trypsin addition. Release of the mature protein was observed following the addition of trypsin. **B**: S453P Proaerolysin in a 50 mM Tris 50 mM NaCL pH 8 buffer was processed with insoluble agarose trypsin beads. Unprocessed and processed toxins were incubated with His-bind resin preloaded with Nickel and incubated at 4°C for 30 minutes to allow binding of the protein and then extensively washed. The bead sample (input) was then split in two: half was treated with 4 M Urea (Urea) and the other half with 250 mM Imidazole (Imid.). After spinning down the beads, the supernatants were analyzed by SDS-PAGE followed by Coomassie blue staining. **C**: Coomassie blue stained gels, as in B, were quantified for 3 independent experiments using ImageJ. Error bars represent standard deviations.

A corollary of the observation that the CTP inhibits oligomerization is that the CTP must be displaced from the mature protein for the process to occur and thus that weaker CTP binding should promote oligomerization. We first tested whether the S453P CTP would be released more readily than the WT CTP. To address this issue, WT and S453P proaerolysins were bound to Nickel charged NTA Surface plasmon resonance (SPR) sensor chips –via the His-tag at the C-terminus of the CTP– and cleavage of the CTP was induced by trypsin addition. After trypsin addition, a strong loss of signal was observed (Figure 9A), presumably corresponding to the release of the mature toxin from the chip-bound CTP. From these curves, we estimated an apparent K_off_ of 4.5.10^−3^±0.6.10^−3^ s^−1^ for WT and 2.6.10^−2^±0.4.10^−2^ s^−1^ for S453P, confirming that the off rate of the S453P CTP was about 10 times higher than that of the WT CTP. These K_off_ values should, however, only be considered in a comparative and not an absolute manner. The structure of aerolysin H132N and the experiments of binding WT aerolysin-CTP to Nickel beads (Figure 1DE) indeed show that the WT CTP does not come off over a period of several hours, which is inconsistent with a K_off_ in the order of 10^−3^ s^−1^. Binding of the CTP to the SPR chip therefore appears to have induced an accelerated release.

That the S453P CTP has a lower affinity for the mature toxin was confirmed by the observation that upon binding of S453P aerolysin-CTP to Nickel beads, about 40% of the total aerolysin was recovered in the unbound fraction (Figure 9BC), i.e. it was released from the bead-bound CTP, whereas less than 10% of the aerolysin was released when performing a similar experiment with the WT toxin (Figure 1DE). Importantly, the CTP-free aerolysin fraction recovered from the S453P-treated beads had the same hemolytic activity as WT proaerolysin treated with trypsin (9±1 wells lysed in 60 min for CTP-free aerolysin (n = 3) and 8±0.5 for trypsin treated WT proaerolysin, see methods). Three important conclusions can be drawn from this observation: 1) the CTP is not required for pore formation, confirming our previous findings [13]; 2) CTP-free aerolysin does not unfold –but might change conformation– since it retains its full activity; 3) CTP-free aerolysin does not undergo unproductive aggregation, thus the role of the CTP is not merely to prevent aggregation of monomers.

**Figure 10:**
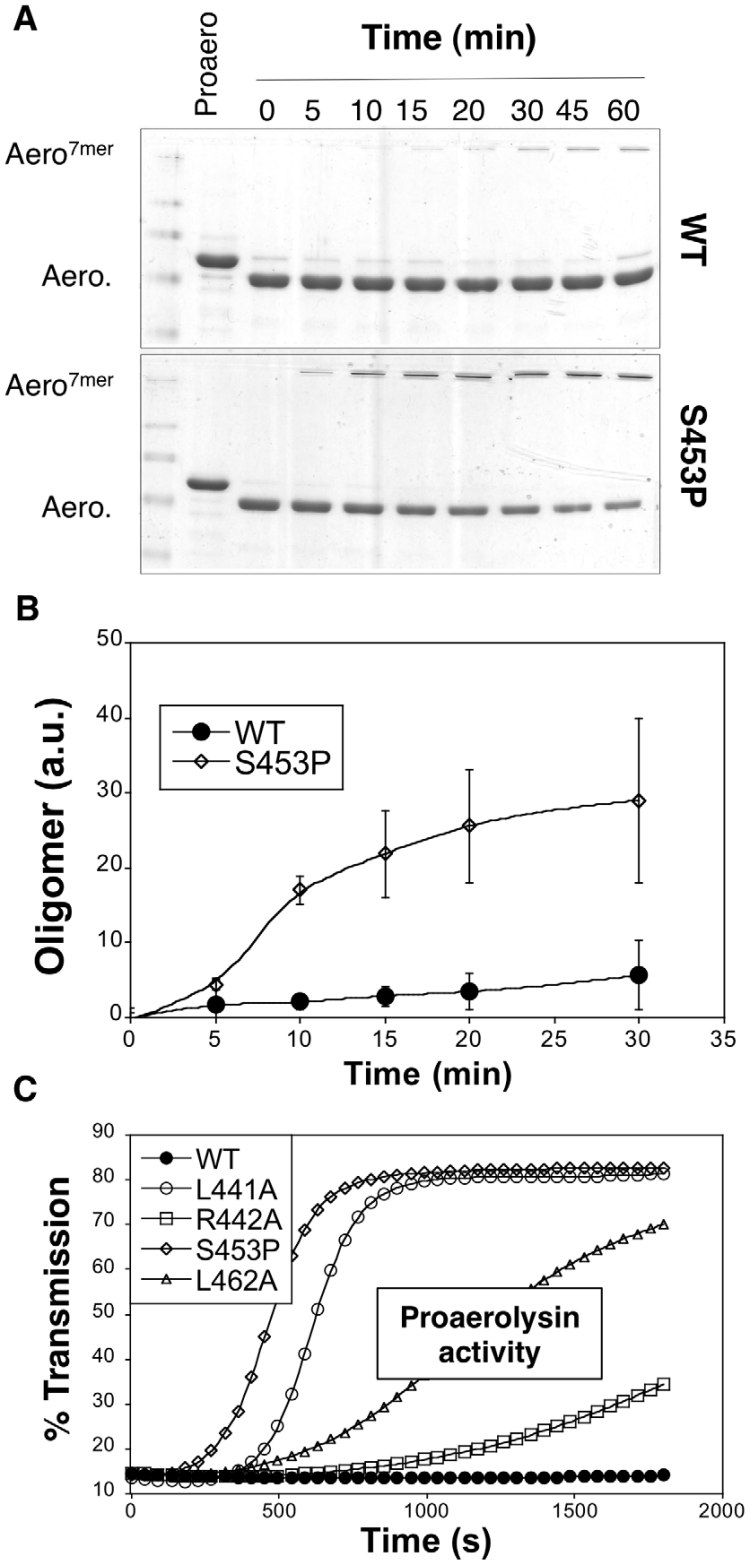
Effect of the S453P mutation on oligomerization and activity. **A**: WT proaerolysin, kept in 20 mM Mes 150 NaCl pH 5, was diluted 2 fold with a 20 mM Tris 150 NaCl pH 8 buffer while S453P proaerolysin, kept in 20 mM Tris 150 NaCl pH 8, was diluted 2 fold with a 20 mM Mes 150 NaCl pH 5 buffer. Following dilution this dilution (final toxin concentration 0.4 mg/ml), the samples were processed on ice with trypsin and then incubated at room temperature to allow oligomerization. Aliquots were removed at different times and the extent of oligomerization was monitored by SDS-PAGE and Coomassie blue staining. **B:** Coomassie blue stained gels, as in A, were quantified for 3 independent experiments using ImageJ. Error bars represent standard deviations. **C**: WT and mutant proaerolysins, in the absence of any protease treatment, were added to erythrocytes at 2 µg/mL and the transmitted light at 600 nm of the sample was followed at room temperature as a function of time. Plots represent the percentage of transmitted light as recorded by the SpectraMax ME as a function of time. This is a representative experiment out of 4 independent experiments.

If CTP release is necessary for oligomerization, then oligomerization should be accelerated when CTP binding is weaker. This is indeed what we observed when comparing oligomerization of WT and S453P: upon trypsin cleavage of S453P proaerolysin, oligomerization occurred faster than for WT (Figure 10AB).

To our surprise, we found that S453P actually already showed some hemolytic activity even in the absence of trypsin cleavage (Figure 10C), which is never observed for WT proaerolysin. This activity was some 15 fold lower than upon trypsin cleavage, yet significant and reproducibly detectable. Hemolytic activity in the proaerolysin form was also observed for the other CTP mutants L441A, R442A and L462A, but to a lesser extent (Figure 10C). These observations show that cleavage of the loop linking the CTP to Domain 4 is not essential and that peptide displacement is sufficient.

## Discussion

Guided by a combination of molecular simulations and *in silico* mutagenesis analysis and using a combination of structural and functional assays on WT and mutant toxins, we show that the CTP is essential for the folding of aerolysin into a soluble toxin. Due to the fact that it promotes folding but is not part of the final active conformation of the protein, i.e. the transmembrane heptameric pore, the CTP qualifies as a chain-linked molecular chaperone [18]. Chaperones comprise both proteins that favor the folding reaction of substrate proteins and proteins that control the quaternary assembly of multisubunit complexes. These two distinct roles can also be found in chain-linked, or intramolecular, chaperones and have been termed type I (folding) and type II (assembly) intramolecular chaperones [18]. Chain-linked chaperones can be short peptides (≈40 residues) or independent folding units. They are often found in secreted or transmembrane proteins, a situation that requires the protein to be translocated across the plasma membrane in prokaryotes (as for proaerolysin) or the ER membrane in eukaryotes. As discussed below, due to the directionality of membrane translocation coupled to protein synthesis, type I intramolecular chaperones are found at the N-terminus of proteins. However, exceptions, such as aerolysin, exist. Indeed, an N-terminal chaperone prevents misfolding *a priori*, while a C-terminal chaperone would act *a posteriori*. In contrast, most documented type II intramolecular chaperones are C-terminal. Irrespective of their localization, chain-linked chaperones should not be part of the final structure, which implies that they must be cleaved off at some point.

One of the earliest and best-characterized examples of a protein with an N-terminal intramolecular chaperone is *Bacillus subtilis* subtilisin, in which the 77 first amino acids fold into a well defined domain promoting the folding of the next 275 residues, acting as a type I chaperone, and is subsequently cleaved off by autoproteolysis [19].

C-terminal intramolecular chaperones have also been described. They are, however, generally of the type II, playing a role in controlling the quaternary assembly of proteins such as tail spikes of bacteriophages or fiber forming collagen [18]. Examples of type I C-terminal chaperones are rare and evidence is circumstantial [20], [21], [22], [23]. Aerolysin thus appears to be the first example of a protein bearing a C-terminal chain-linked chaperone promoting the formation of soluble monomeric subunits and controlling assembly of the active complex, i.e. both type I and type II. The present studies indeed show that the aerolysin CTP acts as a type II chaperone in controlling the onset of heptamerization, a role consistent with its C-terminal location. More unexpectedly, we found that the CTP drives formation of soluble proaerolysin. Mutations in the CTP that affects its structure (S453P) or its binding to Domain 4 (L441A, F457G) indeed lead to aggregation of proaerolysin both *in vivo* and *in vitro*. Moreover aerolysin, devoid of CTP, also aggregated. Importantly, addition in *trans* of a 5 fold molar excess of synthetic CTP allowed partial recovery of activity. Upon CTP release, the trigger for which remains to be established, aerolysin remains folded, possibly with a somewhat different conformation, as illustrated by the full hemolytic activity of CTP-free aerolysin obtained from the S453P mutant. The unaltered activity of CTP-free aerolysin also indicates that the CTP plays a role in the biogenesis of the toxin and does not prevent unproductive aggregation of protein once folded. Altogether, these observations thus classify the aerolysin CTP as a chain-linked intramolecular chaperone.

Our observations clearly indicate that the CTP prevents aggregation of proaerolysin during biosynthetic folding. As mentioned above, and as supported by the ability of the CTP to promote recovery of hemolytic activity upon in vitro folding of aerolysin, the CTP appears to do more than merely preventing aggregation as also suggested by the molecular dynamics studies. Confirming that the CTP promotes the folding of aerolysin and how it does so will require further investigation. Since proaerolysin is translocated from N- to C-terminus when crossing the inner *Aeromonas* membrane, the CTP appears last. In particular, it appears some 250 residues later than some of the residues it interacts with. What is also puzzling is that the CTP is not an independent folding unit that could guide folding of the rest of the protein, as is the case for most type I intramolecular chaperones. Our MD simulations suggest that when released from the protein, as mimicked by the F457G mutation, or when free in solution, the CTP is largely unfolded (Figure S2BC). Therefore the CTP might stabilize folding intermediates. It has been proposed that, as a protein follows its folding landscape, the chaperone domain binds to, stabilizes and increases the population of molecules with native conformations. Thus, as opposed to general chaperones, which are thought to lack any structural information about the protein they fold, dedicated chaperones and possibly the aerolysin CTP could promote folding via conformational selection [24], [25], [26] and thus convey steric information. This hypothesis is consistent with the observation that one segment of Domain 4 with which the CTP interacts in the final structure, residues 269–279, is predicted to be unstructured. Even though largely unfolded, such segments are likely to fluctuate between multiple folded states during short times, one of which could be stabilized by the CTP. A prediction from the conformational selection model for CTP-mediated proaerolysin folding is that folding should be affected by mutations in the CTP. This is indeed what we observed for the mutants suggested by *in silico* alanine scanning mutagenesis and in particular for the S453P and F457A/G mutations.

As mentioned above, the CTP appears to force segment 268–272 into a β-strand conformation. Importantly, this segment is directly followed by the loop in Domain 3 that is to form one of the amphipathic ß-hairpins of the heptameric transmembrane ß-barrel pore (Figure S2A). The ability of the CTP to control the folding state of the underlying β-strands (note that Domain 4 shares multiple β-strands with Domains 3 and 2) suggests that the peptide also acts as a switch to control the pore formation process. Our observations indeed show that CTP release promotes oligomerization and that the CTP is not part of the final pore. Future studies will address what triggers release of the CTP. Our preliminary observations indicate that specific detergents can displace the CTP, consistent with the importance of hydrophobic interactions in CTP binding and suggesting that the target cell membrane may play a role. Future studies will also address whether CTP release triggers partial unfolding of Domain 4 and whether these changes propagate to Domain 3 helping overcome the energy barrier that leads to formation of the heptamer, the most thermodynamically stable conformation [14].

## Methods

### Proaerolysin production

Proaerolysin WT and mutants were expressed using a pET22b vector (Novagen), which allows periplasmic expression of the toxin with a His6 tag on the C-terminus, as described [7]. Mutagenesis was carried out using the Quick Change Kit (Stratagene). Briefly, BL21 [DE3] pLysS *E. coli* harboring the WT or mutant aerolysin expression plasmid were grown at 37°C to an OD_600_ of 0.6. IPTG (0.25 mM) was added and cultures were shifted to 16°C for protein production. Cells were harvested after ≈2 hrs (reaching an OD_600_ = 1.2). Periplasmic fractions were isolated by resuspending cells in T Buffer (0.1 M Tris-HCl pH 8.0, 18% sucrose) containing 5 mM EDTA and 0.2 mg/mL lysozyme. After agitation for 30 min at 4°C, the periplasm and spheroplasts were separated by centrifugation. For purification, the supernatant was further ultracentrifuged (100 000 x g for 2 h at 4°C), filtered (0.45 µm) and dialyzed against 20 mM sodium phosphate buffer pH 7.4, 0.5 M NaCl and loaded on a 1 mL HiTrap chelating column (Amersham Pharmacia Biotech) running on an AKTA^TM^ prime FPLC workstation. The protein was eluted in a 20 mM sodium phosphate buffer pH 7.4, 0.5 M NaCl buffer with a linear gradient of imidazole (0–0.5 M). Finally, fractions containing the protein were dialyzed against 20 mM MES buffer pH5, 150 mM NaCl before snap freezing and storing at −80°C. Protein concentration was determined by O.D._280_ measurements using an estimated ε≈13.05·10^4^ M^−1^·cm^−1^. The S453P single point mutant was dialysed into 20 mM Tris 150 mM NaCl pH 8 following purification due to its tendancy to oligomerizes unprocessed when dialyzed into the MES pH 5 buffer.

### Trypsin cleavage of proaerolysin

Unless specified, processing of proaerolysin was performed by addition of 1/100 (weight/weight) of soluble trypsin (Sigma) and incubation for 10 min at room temperature. Where specified, the proaerolysin containing solution of 20 mM MES buffer pH5, 150 mM NaCl was adjusted to pH 8 by addition of 1 M Tris buffer pH 8.7 to avoid oligomerization [11]. Pre-washed trypsin immobilized on agarose beads (Sigma) was added to the proaerolysin sample at 4°C and incubation was allowed to proceed on a rotary shaker for 2 hrs. The trypsin agarose beads were removed by centrifugation at 7000 rpm in a tabletop Eppendorf centrifuge. The degree of activation was assessed by SDS-PAGE and Coomassie blue staining.

### Hemolytic activity

Activity of aerolysin was defined by its ability to lyse red blood cells. Serial dilutions of aerolysin starting at 20 µg/ml were incubated with a 0.5% solution of red blood cells in a 96 well plate. Activity was either recorded as number of wells fully lysed in 60 min at room temperature [7] or as the transmitted lightof the erythrocyte suspension monitored at 600 nm as a function of time in a given well using an automated 96 well plate reader at 37°C.

### Peptide inhibition of oligomerization

Proaerolysin at a concentration of 0.4 mg/mL was submitted to proteolysis with trypsin bound to agarose beads as described above. A 5 fold mol/mol excess of synthetic propeptide (EzBiolabs), control peptide, or an equal volume of buffer, was added to the sample. To initiate the oligomerization process (which requires a pH<8 and is promoted by low salt), the sample was dialyzed at 4°C against 10 mM Hepes buffer pH 7, 10 mM NaCl for 2–4 hours. The dialysis molecular weight cut off was 3.5 kDa, unless specified otherwise. Aliquots were removed at different time points and subjected to SDS-PAGE.

### Unfolding, circular dichroism and tryptophan fluorescence measurements

Tryptophan fluorescence was measured as described [14] using a SpectraMax M2e spectrofluorimeter. Circular dichroism (CD) measurements were performed at 20°C using a Jasco J815 spectrometer using quartz cells of 0.01 cm path length [13]. Spectra between 190 and 250 nm were recorded in 20 mM Hepes buffer pH 8, 50 mM NaF at protein concentrations between 0.1–0.3 mg/mL.

For unfolding, proaerolysin WT or mutant or aerolysin was incubated in 4 M urea (see Protocol S1). Refolding was triggered by 1:10 dilution into urea free buffer. The refolding reaction was assessed by circular dichroism or hemolytic activity. The buffer blank solution was obtained by dilution of the respective buffers.

### Structural models and molecular dynamics simulations

WT proaerolysin in its dimeric form has been crystallized with a resolution of 2.8 Å (entry 1PRE in protein databank). In this crystal structure, two loops located on top of Domain 4 proved too flexible to be crystallized, namely residues 207 to 211 and 423 to 439. A crystal structure of dimeric proaerolysin mutant Y221G has been obtained with a higher resolution of 2.2 Å (entry 3C0N in protein databank). In this crystal, residues 207 to 211 could be mapped in the crystal structure but loop 423 to 439 is still missing. This loop connects the CTP to the rest of the protein, and contains the site where cleavage takes place during aerolysin activation (420–427).

A model of wild-type aerolysin with the propeptide bound (labeled aerolysin-WT) to use in molecular dynamics simulations was obtained by using 3C0N structure, and mutating residue 221 back to tyrosine using 1PRE as a structural template (wild-type rebuilding did not caused any steric problem since 3C0N and 1PRE were virtually identical). We assumed that this model would mimic the aerolysin structure after cleavage, i.e. C-terminal propeptide no longer covalently connected to the protein, but still bound to it. In fact, this model is structurally equivalent to the cleaved aerolysin H132N X-ray structure showed in this work. We modeled active aerolysin (labeled WT) by removing the propeptide from the previous model. Mutation F457G has been performed by removing the Phe-457 side-chain.

Aerolysin contains six histidines. Their protonation state at physiological pH has been defined by the presence of proton donors and acceptors in their neighborhood in the crystal structure. We concluded that in H107, H121, H132, H186 and H332 Nε atom is protonated, whereas in H341 Nδ is protonated. These model systems were solvated in a rectangular box of pre-equilibrated TIP3P water molecules, and their total charge was neutralized by the addition of Na^+^ and Cl^−^ counterions. Molecular dynamics has been performed for aerolysin with CTP (labeled Aero-CTP), without CTP (labeled Aero) and mutation F457G using the Amber parm99sb force field [27] on NAMD molecular dynamics engine [28], using the SHAKE algorithm on all the bonds, and Particle-mesh Ewald for treating the electrostatic interactions in periodic boundary conditions [29]. We used an integration time step of 2 fs. The systems were energy-minimized by means of 1000 conjugate gradient steps, and subsequently gradually heated from 0 to 300 K in 1 ns at 1 atm. Simulations were run in the NPT ensemble at 1 atm and 300 K. Temperature was controlled by means of Langevin forces, using a damping constant of 1 ps^-1^.

Preliminary results confirmed that Domain 1 is bulky and, being connected by a long random coil to the large lobe (Figure 1A), extremely flexible with respect to the rest of the protein. Since this resulted in no influence on the structure of the other domains, we decided to remove it in order to reduce the system size and therefore speed up the remaining computation. All simulations were run for at least 200 ns. RMSD of MD simulations showed that every system equilibrated in around 10 ns.

Alanine scanning was performed on the single 200 ns molecular dynamics trajectory of CTP-WT system. A subset of 200 decorrelated frames (one every 10 ns) was extracted. On this subset, we calculated binding free energies of Ala mutant species using the MM-PBSA method, as implemented in the AMBER molecular dynamics package (24). The Poisson-Boltzmann method was used to compute the electrostatic contribution to the solvation free energy. Ionic strength molarity was set to 0.1 M, the protein dielectric constant to 1, and the solvent to 80. Every residue being part of the CTP, excluding glycines and prolines, was scanned. These residues play a major role in the determination of strand flexibility, thus the alanine scanning is known to perform poorly. The MM-PBSA method was also used to estimate the binding free energy of WT and F457G mutated CTP to domain 4. For both these measures, 200 decorrelated frames extracted from aero-CTP and F547G MD simulations were used, respectively. Analysis of MD trajectories, as well as rendering of protein structures, has been performed using VMD [30].

## Supporting Information

Figure S1 **A:** Sequence of the pre-pro-aerolysin used in this study. The periplasmic secretion motif is colored black and the different domains of the proteins are color-coded cyan (Domain 1), red (Domain 2), green (Domain 3) and orange (Domain 4). The CTP is shown in blue with the residues described in this study shown in bold. The flexible linker connecting the CTP to the Domain 4 of the protein is shown in bold. **B**: Two points of view of proaerolysins with Ile-445 mutated to cysteine to which IEADANS has been attached.
(TIF)

Figure S2 **A**: Disorder predictions performed using eight different prediction algorithms on the proaerolysin sequence. In red, the region spanning Gln-268 to Arg-282 was predicted by at least six algorithms to be disordered. **BC**: Secondary structure along a 195 ns MD simulation in explicit water of CTP (from crystal structure 1PRE). **B**: Time vs CTP residues. A black area indicates that a residue is part of an α-helix at a given time. On the left side is a cartoon representation of the CTP structure with arrows depicting beta-strands and rectangle representing alpha helix. **C**: MD snapshots taken every 50 ns. Snapshot at time zero is colored according to the percentage of time every residue spends being part of an α-helix: red areas are mostly helical, blue areas are not. Residues 449 to 454 (i.e. the alpha helix in the crystal structure) spend ∼70% of the time in α-helix, and appear to be the only structured region of CTP.
(TIF)

Figure S3 **AB**: Pro (black) and activated (grey) aerolysin samples (20 µM) were incubated with different concentrations of GdnHCl (**A**) or urea (**B**) for 2 hrs. Activation was performed prior to unfolding with trypsin agarose beads that were subsequently removed. Fluorescence was measured with an excitation wavelength of 280 nm and the fluorescence emission intensity ratio at 345/315 nm was determined and plotted as a function of urea concentration. **C**: Urea unfolding curves of WT and different CTP mutant proaerolysins described in this study (as in **A**).
(TIF)

Video S1 Molecular dynamics simulation of proaerolysin F457G. Result of a molecular dynamics simulation of proaerolysin F457G. The secondary structure of every frame, as calculated by the DSSP algorithm, is represented in the cartoon with different colors: yellow is beta sheet; white and cyan are random coil; purple, blue, and red are alpha helix). Mutation F457G, located on the CTP, has a destabilizing effect. Indeed, the CTP loses most of its secondary structure and begins to disconnect from Domain 4. On Domain 4 an unfolding similar to the one observed in the absence of CTP can be detected. The movie was rendered using the VMD software.
(MP4)

Video S2 Molecular dynamics simulation of aerolysin WT without the CTP. Result of a molecular dynamics simulation of aerolysin WT with CTP manually removed. The secondary structure of every frame, as calculated by the DSSP algorithm, is represented in the cartoon with different colors: yellow is beta sheet; white and cyan are random coil; purple and blue are alpha helix. Two strands unfold from Domain 4 in the direction of the loop region in Domain 3.
(MP4)

Protocol S1 Are described the protocols for Crystallization, structure determination and refinement of the H132N aerolysin mutant, Unfolding and refolding measurements, Disorder prediction algorithms
(DOC)
