# Supplementary figures and images for: Dual Chaperone Role of the C-Terminal Propeptide in Folding and Oligomerization of the Pore-Forming Toxin Aerolysin
Source: PLoS Pathog. 2011 Jul 14;7(7):e1002135. doi: 10.1371/journal.ppat.1002135 (PMC3136475; doi:10.1371/journal.ppat.1002135)

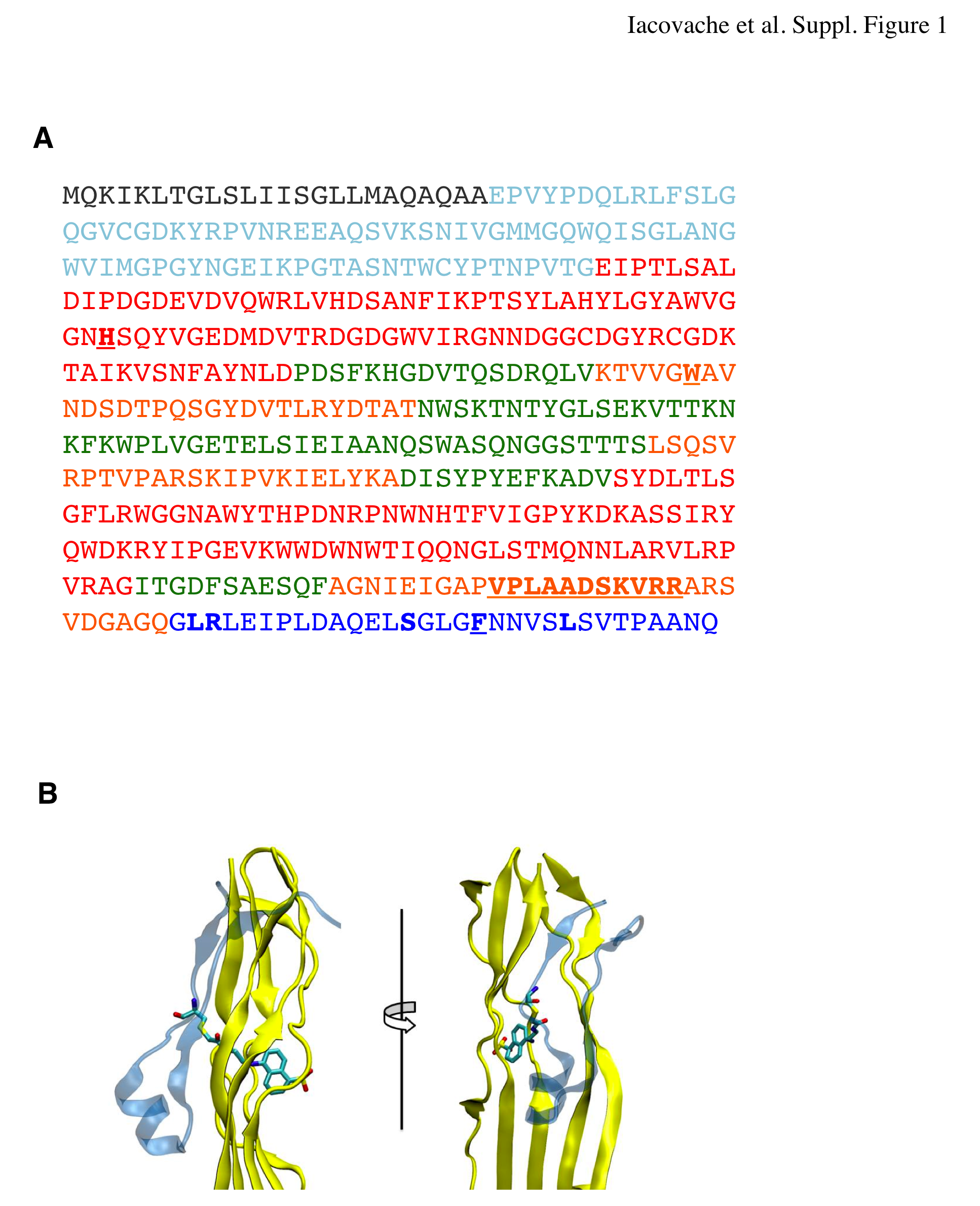

Supplement: Figure S1 — A: Sequence of the pre-pro-aerolysin used in this study. The periplasmic secretion motif is colored black and the different domains of the proteins are color-coded cyan (Domain 1), red (Domain 2), green (Domain 3) and orange (Domain 4). The CTP is shown in blue with the residues described in this study shown in bold. The flexible linker connecting the CTP to the Domain 4 of the protein is shown in bold. B: Two points of view of proaerolysins with Ile-445 mutated to cysteine to which IEADANS has been attached. (TIF) [file ppat.1002135.s001.tif]

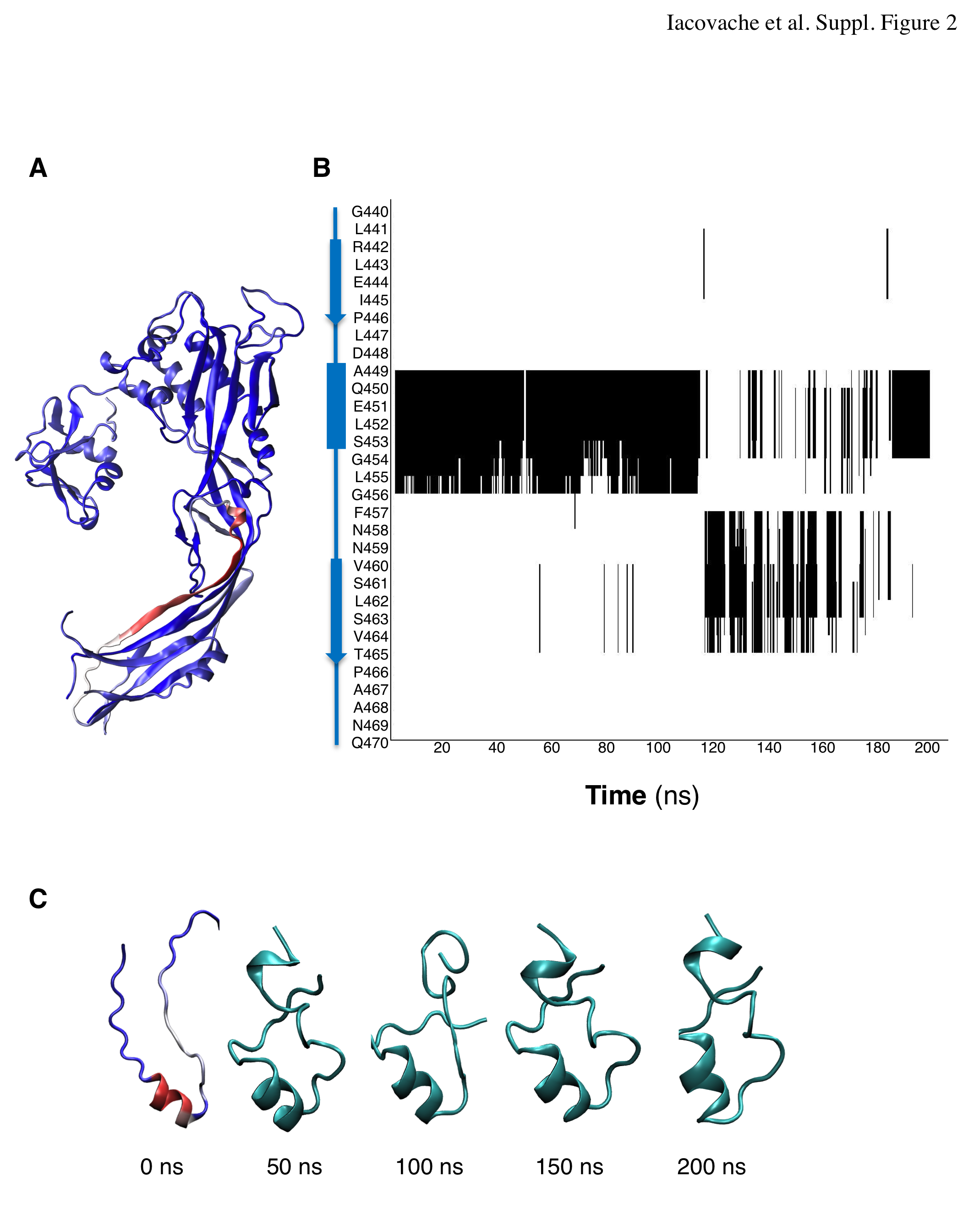

Supplement: Figure S2 — A: Disorder predictions performed using eight different prediction algorithms on the proaerolysin sequence. In red, the region spanning Gln-268 to Arg-282 was predicted by at least six algorithms to be disordered. BC: Secondary structure along a 195 ns MD simulation in explicit water of CTP (from crystal structure 1PRE). B: Time vs CTP residues. A black area indicates that a residue is part of an α-helix at a given time. On the left side is a cartoon representation of the CTP structure with arrows depicting beta-strands and rectangle representing alpha helix. C: MD snapshots taken every 50 ns. Snapshot at time zero is colored according to the percentage of time every residue spends being part of an α-helix: red areas are mostly helical, blue areas are not. Residues 449 to 454 (i.e. the alpha helix in the crystal structure) spend ∼70% of the time in α-helix, and appear to be the only structured region of CTP. (TIF) [file ppat.1002135.s002.tif]

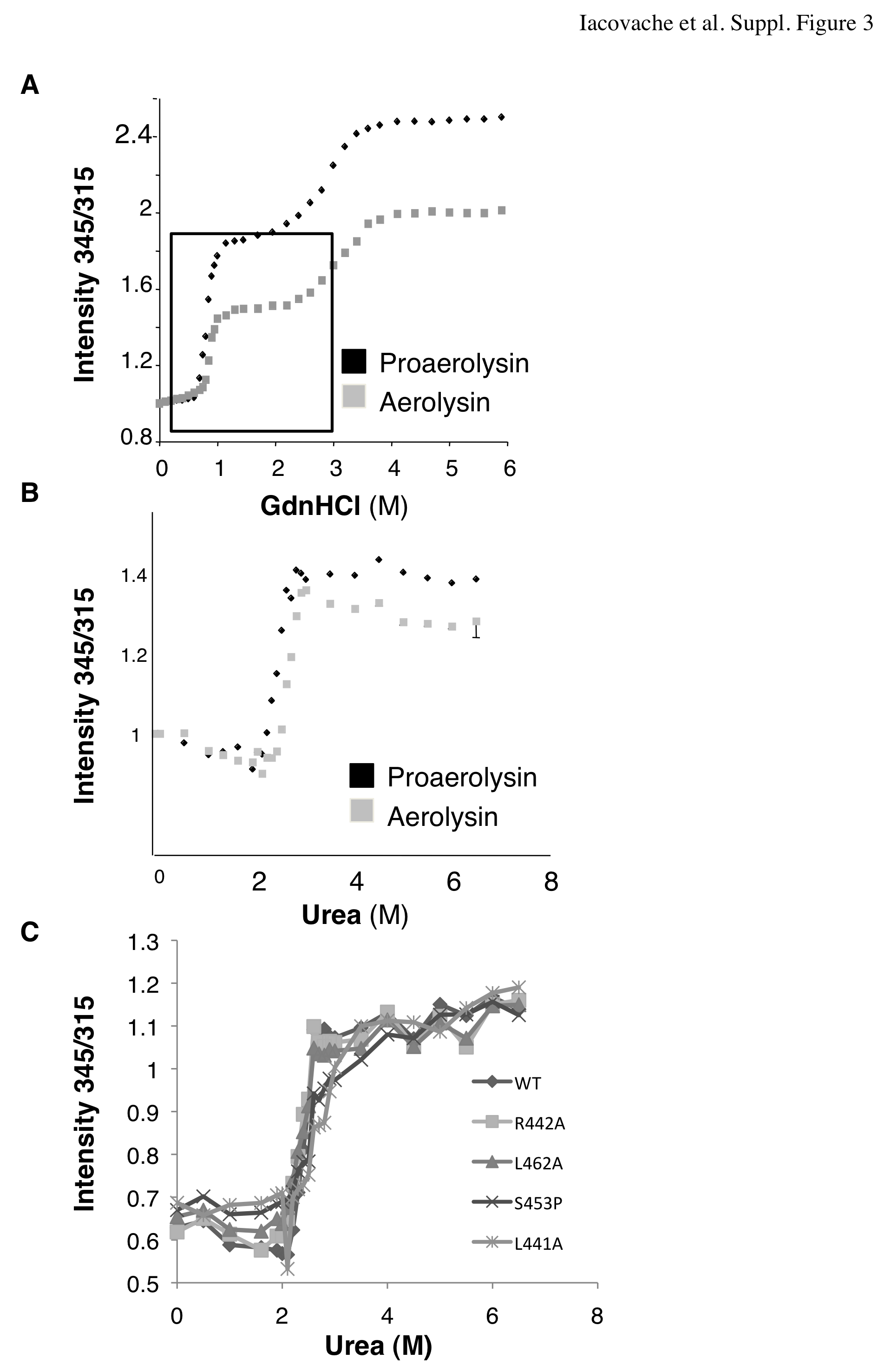

Supplement: Figure S3 — AB: Pro (black) and activated (grey) aerolysin samples (20 µM) were incubated with different concentrations of GdnHCl (A) or urea (B) for 2 hrs. Activation was performed prior to unfolding with trypsin agarose beads that were subsequently removed. Fluorescence was measured with an excitation wavelength of 280 nm and the fluorescence emission intensity ratio at 345/315 nm was determined and plotted as a function of urea concentration. C: Urea unfolding curves of WT and different CTP mutant proaerolysins described in this study (as in A). (TIF) [file ppat.1002135.s003.tif]
