# Supplementary material for: Dual Chaperone Role of the C-Terminal Propeptide in Folding and Oligomerization of the Pore-Forming Toxin Aerolysin
Source: PLoS Pathog. 2011 Jul 14;7(7):e1002135. doi: 10.1371/journal.ppat.1002135 (PMC3136475; doi:10.1371/journal.ppat.1002135)
Supplement: Protocol S1 — Are described the protocols for Crystallization, structure determination and refinement of the H132N aerolysin mutant, Unfolding and refolding measurements, Disorder prediction algorithms (DOC) [file ppat.1002135.s006.doc]

**Protocole S1**

**Crystallization, structure determination and refinement of the H132N aerolysin mutant**

Purified H132N proaerolysin mutant was subjected to proteolysis by trypsin as described [1]. The processed toxin was then dialyzed overnight at 4°C in 20mM HEPES buffer pH 7.4, 150mM NaCl and subjected to gel filtration on a Superdex^TM^ 200 10/300 GL column (GE Healthcare). The purified protein was concentrated with a 30 kDa molecular weight cut off Vivaspin centrifugal concentrator (VivaScience, Sartorius Group) to a concentration of 5 mg/ml at 4°C. Crystals of H132N were grown at 18°C using the hanging-drop vapor-diffusion technique. The best crystals of the activated H132N mutant grew within one week in 24%-28% pentaerythritol propoxylate (5/4 PO/OH), 50mM sodium acetate pH 5.4. The crystal exhibited a space group *C*2 with unit cell parameters *a* = 95.98 Å, *b* 69.31 = Å, *c* = 165.23 Å, b = 109.01° ({Pernot, 2010 #4234} Records of the X-ray diffraction data were performed at 100K. The crystals were briefly plunged in cryoprotectant with the same composition as the mother liquor but with increased pentaerythritol propoxylate (5/4 PO-OH) (to 32% for H132N crystals). The diffraction data sets were collected on the beam line x06sa at the Swiss Light Source, Villigen, Switzerland. Raw diffraction images were indexed and integrated with MOSFLM [2] and scaled with SCALA [3] within the CCP4 program.

The structure of the activated H132N mutant was determined by molecular replacement with the program PHASER [4] using wild-type proaerolysin (PDB accession code 1PRE ; [5]) as a search model. Before starting the molecular replacement procedure the CTP was removed from the initial model (residues L441-Q470). The histidine residue at position 132 was retained. The two solutions that resulted were employed in the refinement procedure performed with CNS 1.1 [6]. Refinement sessions were iteratively interchanged with manual building done with the program O [7]. At the end of the refinement, a translation-libration-screw (TLS) refinement was carried out with the program PHENIX [8]. Each molecular chain in the asymmetric unit was partitioned in nine TLS groups. The choice of the TLS groups was done using the TLSMD web server [9]. Final manual building corrections were done with the program COOT [10]. The geometry of the two final models was checked with MolProbity [11]. Table 1 contains the final crystallographic refinement data.

The coordinates and structure factors are deposited in the Protein Data Bank under the accession codes 3G4O and sf3g4o for H132N and 3G4N and sf3g4n for H132D.

**Table S1:** **Statistics of the refinement** **of the H132N structure**

| ***Refinement*** | **SLS - x06sa** |
| --- | --- |
| Crystal | H132N |
| Resolution | 55.5 - 2.3 |
| No. of reflections | 43641 |
| No. of omitted reflections | 2186 |
| No. of protein residues | 890 |
| No. of water molecules | 187 |
| *R* (%) / *R*_free_ (%) | 20.5 / 26.0 |
| Mean on *B*-factors (Å^2^) |  |
| Protein | 57.7 |
| Solvent | 45.5 |
| r.m.s.d. from ideal geometry |  |
| Bond length (Å) | 0.005 |
| Bond angles (°) | 0.908 |

**Unfolding and refolding measurements**

Urea (U) or Guanidinium hydrochloride (GdnHCl) stock solutions at 10 M and 8 M respectively were prepared fresh [12] and the pH was adjusted to 8. Twenty microliters of concentrated (0.2 – 1 mg/ml) pro or mature aerolysin was adjusted to urea concentrations ranging from 0 to 8 M and allowed to reach equilibrium. Unfolding was monitored by measuring the fluorescence intensity ratio at 345/315nm in a SpectraMax M2e plate reader. The corresponding buffer was subtracted from each measurement.

**Disorder prediction algorithms**

The following eight disorder prediction algorithms were used: RONN [13], DisEMBL1.5 [14], PreLink [15], DripPred [16], OnDCRF [17], GlobPlot [18], PrDOS [19], IUPRED [20]. Predicted values, being globally consistent, were averaged and reported in Supp. Information.

**Supplementary references**

1. Pernot L, Schiltz M, van der Goot FG (2010) Preliminary crystallographic analysis of two oligomerization-deficient mutants of the aerolysin toxin, H132D and H132N, in their proteolyzed forms. Acta Crystallogr Sect F Struct Biol Cryst Commun 66: 1626-1630.

2. Leslie AG (2006) The integration of macromolecular diffraction data. Acta Crystallogr D Biol Crystallogr 62: 48-57.

3. Evans P (2006) Scaling and assessment of data quality. Acta Crystallogr D Biol Crystallogr 62: 72-82.

4. McCoy AJ, Grosse-Kunstleve RW, Storoni LC, Read RJ (2005) Likelihood-enhanced fast translation functions. Acta Crystallogr D Biol Crystallogr 61: 458-464.

5. Parker MW, Buckley JT, van der Goot FG, Tsernoglou D (1997) Structure and assembly of the channel-forming *Aeromonas* toxin aerolysin. book.

6. Brunger AT, Adams PD, Clore GM, DeLano WL, Gros P, et al. (1998) Crystallography & NMR system: A new software suite for macromolecular structure determination. Acta Crystallogr D Biol Crystallogr 54: 905-921.

7. Jones TA, Zou JY, Cowan SW, Kjeldgaard M (1991) Improved methods for building protein models in electron density maps and the location of errors in these models. Acta Crystallogr A 47 ( Pt 2): 110-119.

8. Adams PD, Grosse-Kunstleve RW, Hung LW, Ioerger TR, McCoy AJ, et al. (2002) PHENIX: building new software for automated crystallographic structure determination. Acta Crystallogr D Biol Crystallogr 58: 1948-1954.

9. Painter J, Merritt EA (2006) Optimal description of a protein structure in terms of multiple groups undergoing TLS motion. Acta Crystallogr D Biol Crystallogr 62: 439-450.

10. Emsley P, Cowtan K (2004) Coot: model-building tools for molecular graphics. Acta Crystallogr D Biol Crystallogr 60: 2126-2132.

11. Davis IW, Leaver-Fay A, Chen VB, Block JN, Kapral GJ, et al. (2007) MolProbity: all-atom contacts and structure validation for proteins and nucleic acids. Nucleic Acids Res 35: W375-383.

12. van der Goot FG, Ausio J, Wong KR, Pattus F, Buckley JT (1993) Dimerization stabilizes the pore-forming toxin aerolysin in solution. The Journal of Biological Chemistry 268: 18272-18279.

13. Yang ZR, Thomson R, McNeil P, Esnouf RM (2005) RONN: the bio-basis function neural network technique applied to the detection of natively disordered regions in proteins. Bioinformatics 21: 3369-3376.

14. Linding R, Jensen LJ, Diella F, Bork P, Gibson TJ, et al. (2003) Protein disorder prediction: implications for structural proteomics. Structure 11: 1453-1459.

15. Coeytaux K, Poupon A (2005) Prediction of unfolded segments in a protein sequence based on amino acid composition. Bioinformatics 21: 1891-1900.

16. MacCallum RM Order/Disorder Prediction With Self Organising Maps.

17. Wang L, Sauer UH (2008) OnD-CRF: predicting order and disorder in proteins using [corrected] conditional random fields. Bioinformatics 24: 1401-1402.

18. Linding R, Russell RB, Neduva V, Gibson TJ (2003) GlobPlot: Exploring protein sequences for globularity and disorder. Nucleic Acids Res 31: 3701-3708.

19. Ishida T, Kinoshita K (2007) PrDOS: prediction of disordered protein regions from amino acid sequence. Nucleic Acids Res 35: W460-464.

20. Dosztanyi Z, Csizmok V, Tompa P, Simon I (2005) The pairwise energy content estimated from amino acid composition discriminates between folded and intrinsically unstructured proteins. J Mol Biol 347: 827-839.
